# Supplementary material for: Emerging Resistance of Neglected Tropical Diseases: A Scoping Review of the Literature
Source: Int J Environ Res Public Health. 2019 May 31;16(11):1925. doi: 10.3390/ijerph16111925 (PMC6603949; doi:10.3390/ijerph16111925)

Supplementary Materials S1
Flow charts of studied NTDs and their drugs to treat

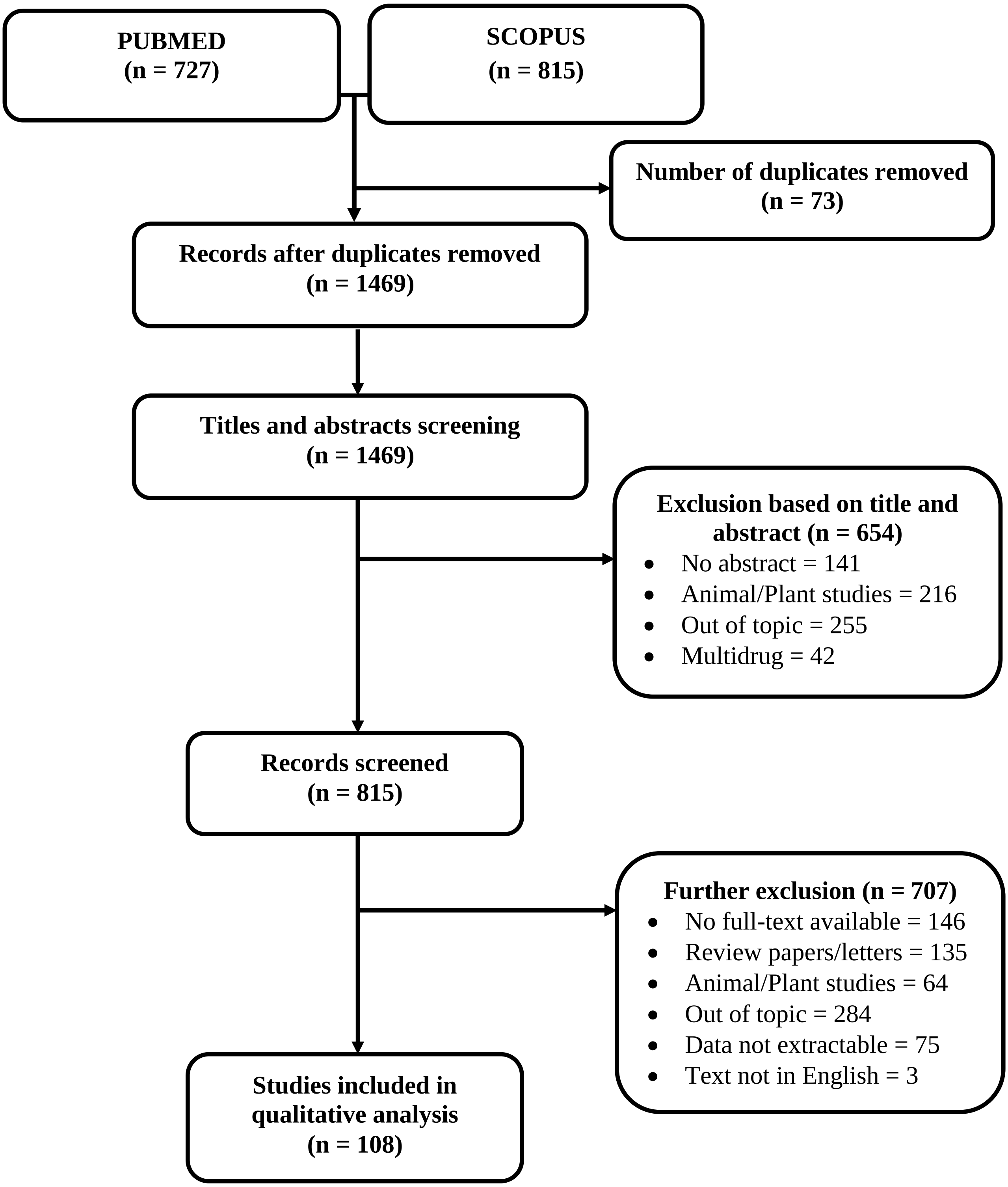

DISEASE: Chagas disease
DRUG: Nifurtimox

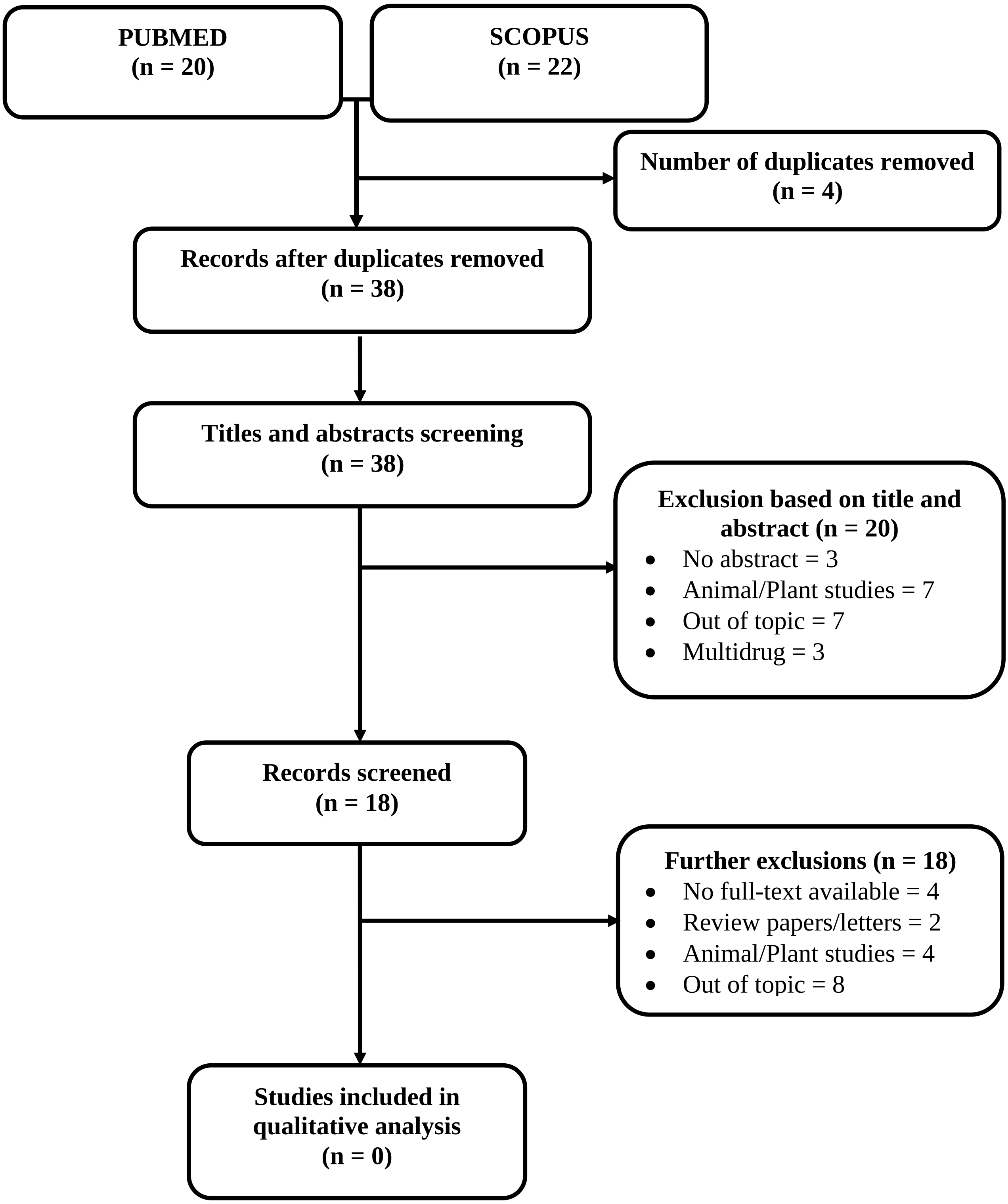

DISEASE: Human African Trypanosomiasis
DRUG: Suramin

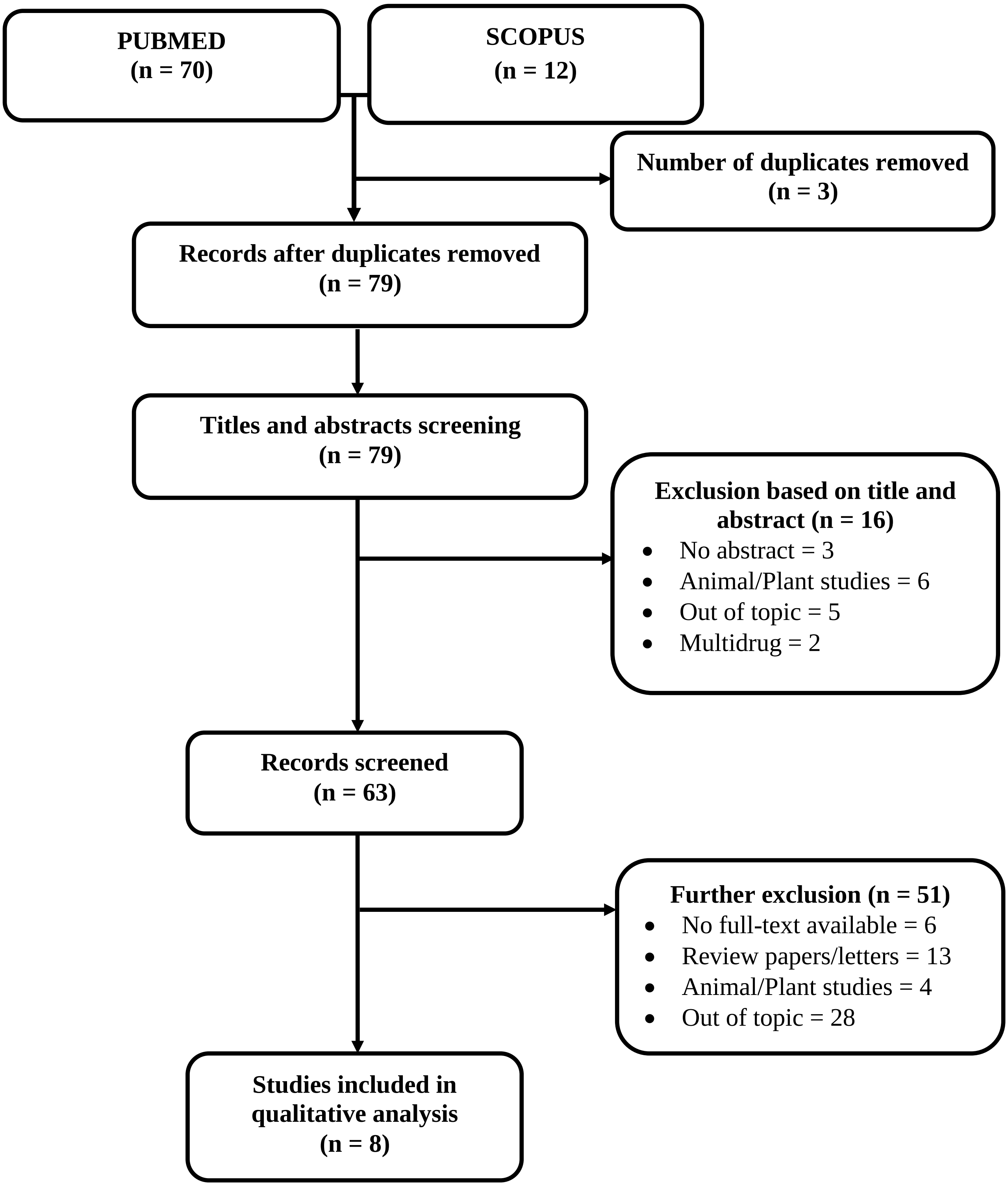

DISEASE: Human African Trypanosomiasis
DRUG: Eflornithine

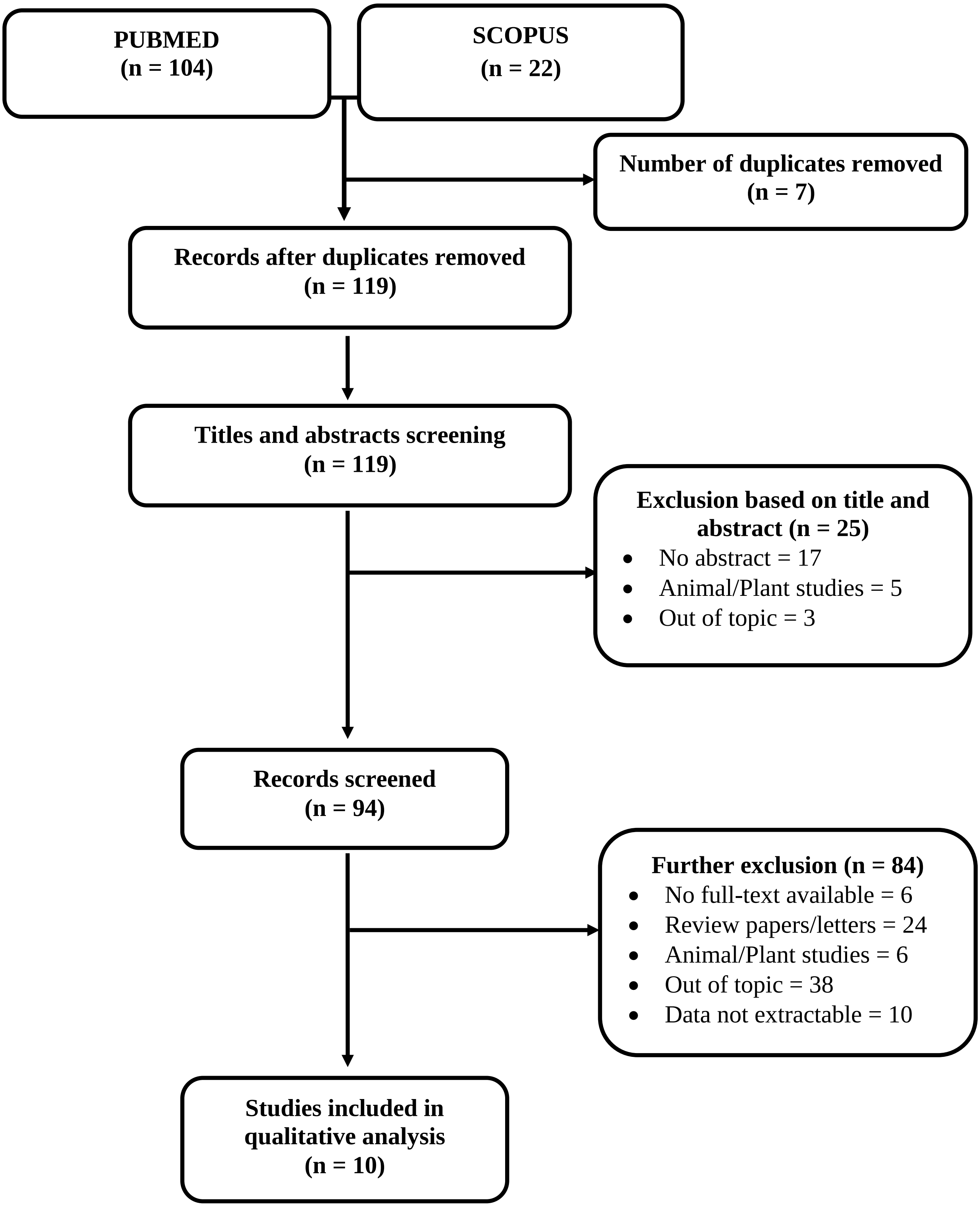

DISEASE: Human African Trypanosomiasis
DRUG: Melarsoprol

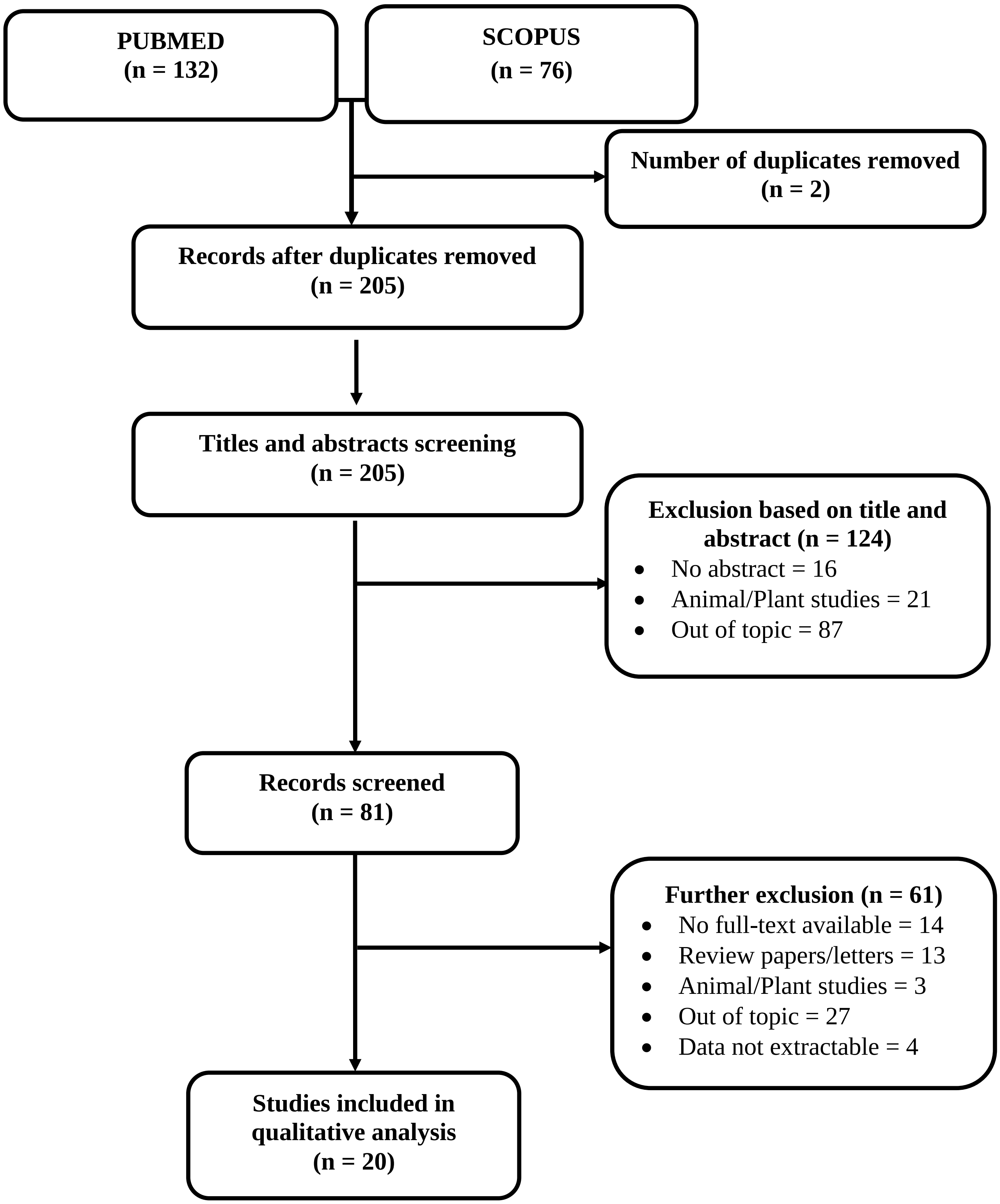

DISEASE: Human African Trypanosomiasis
DRUG: Pentamidine

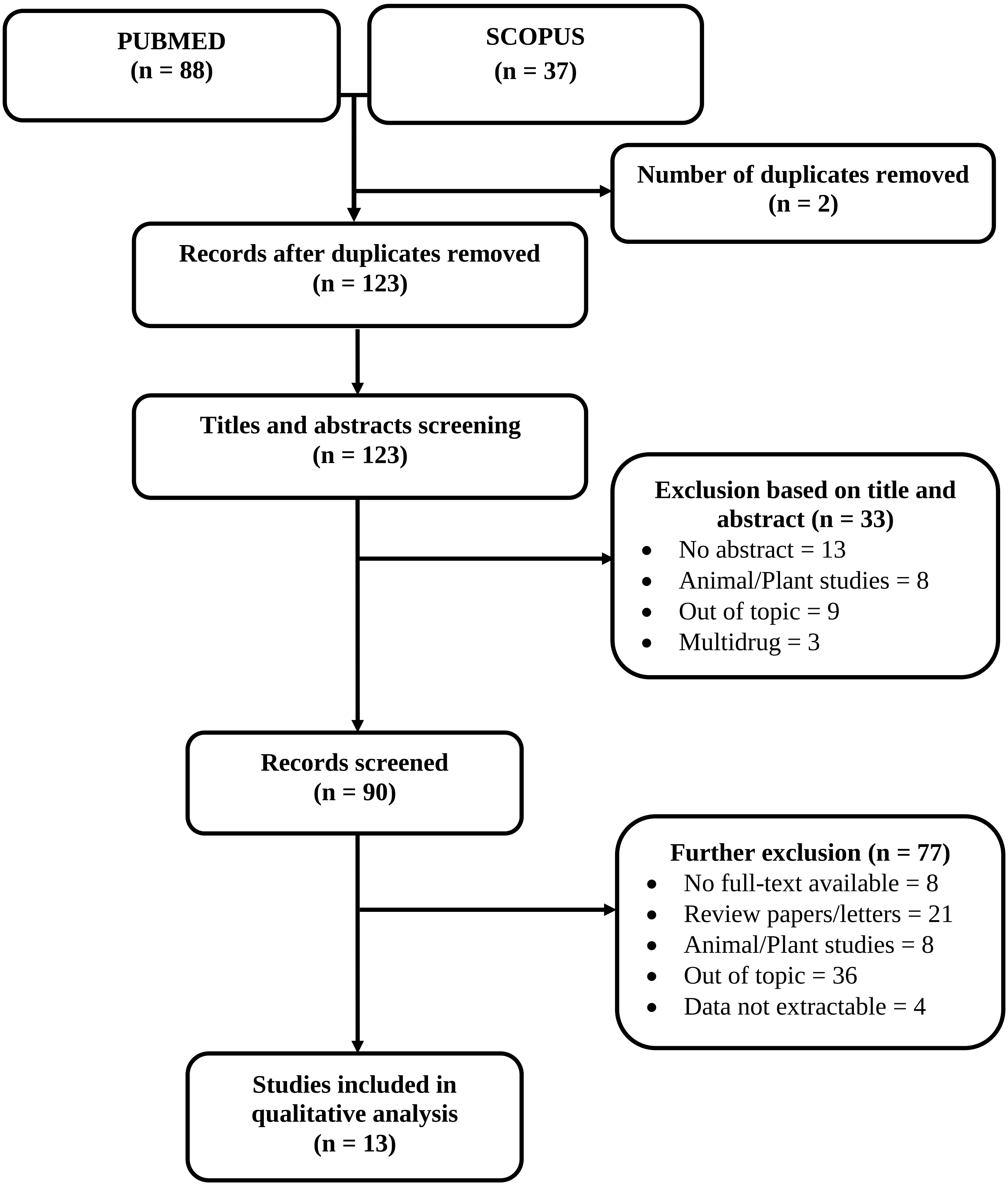

DISEASE: Leishmaniasis
DRUG: Amphotericin B

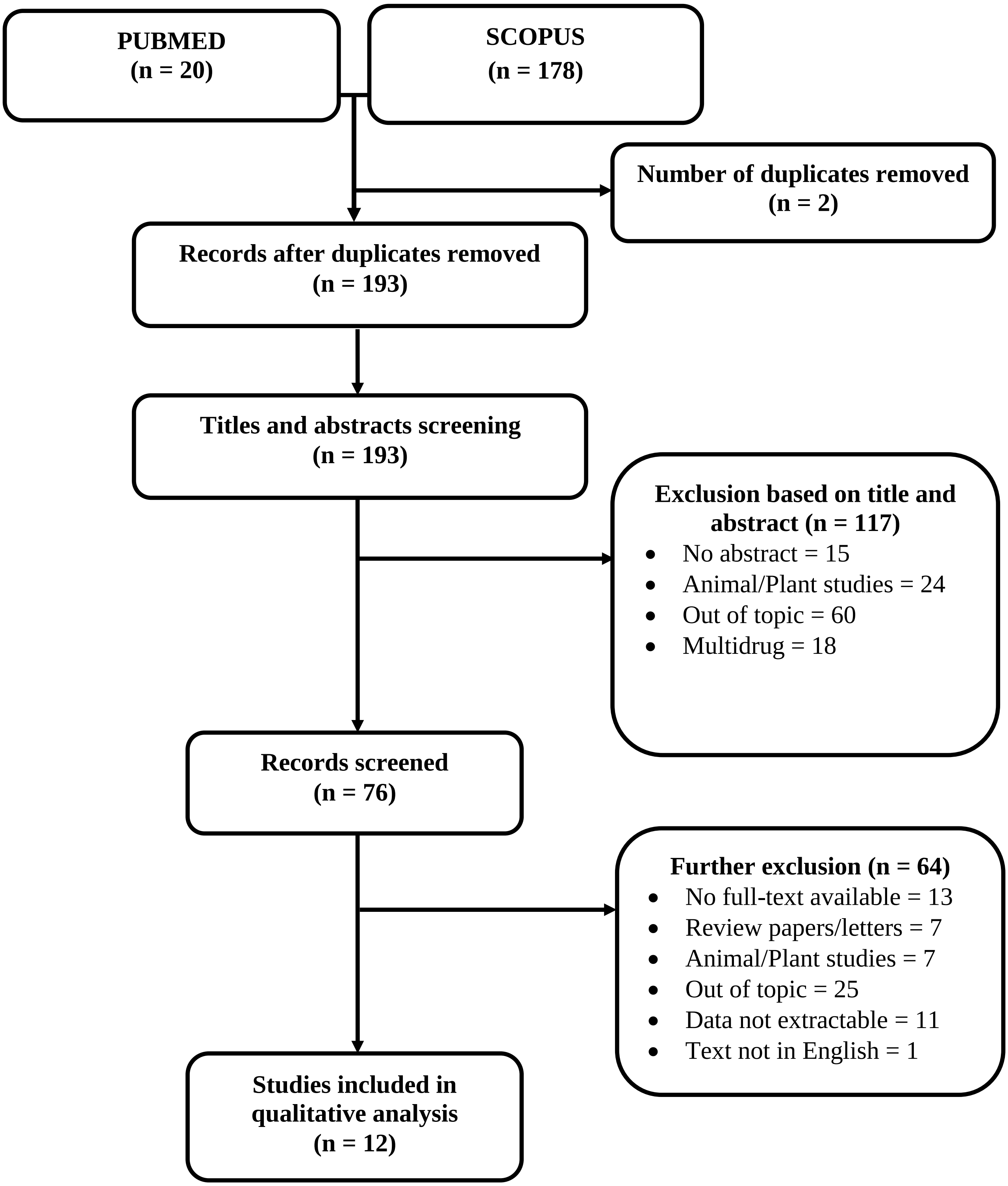

DISEASE: Leprosy
DRUG: Rifampicin

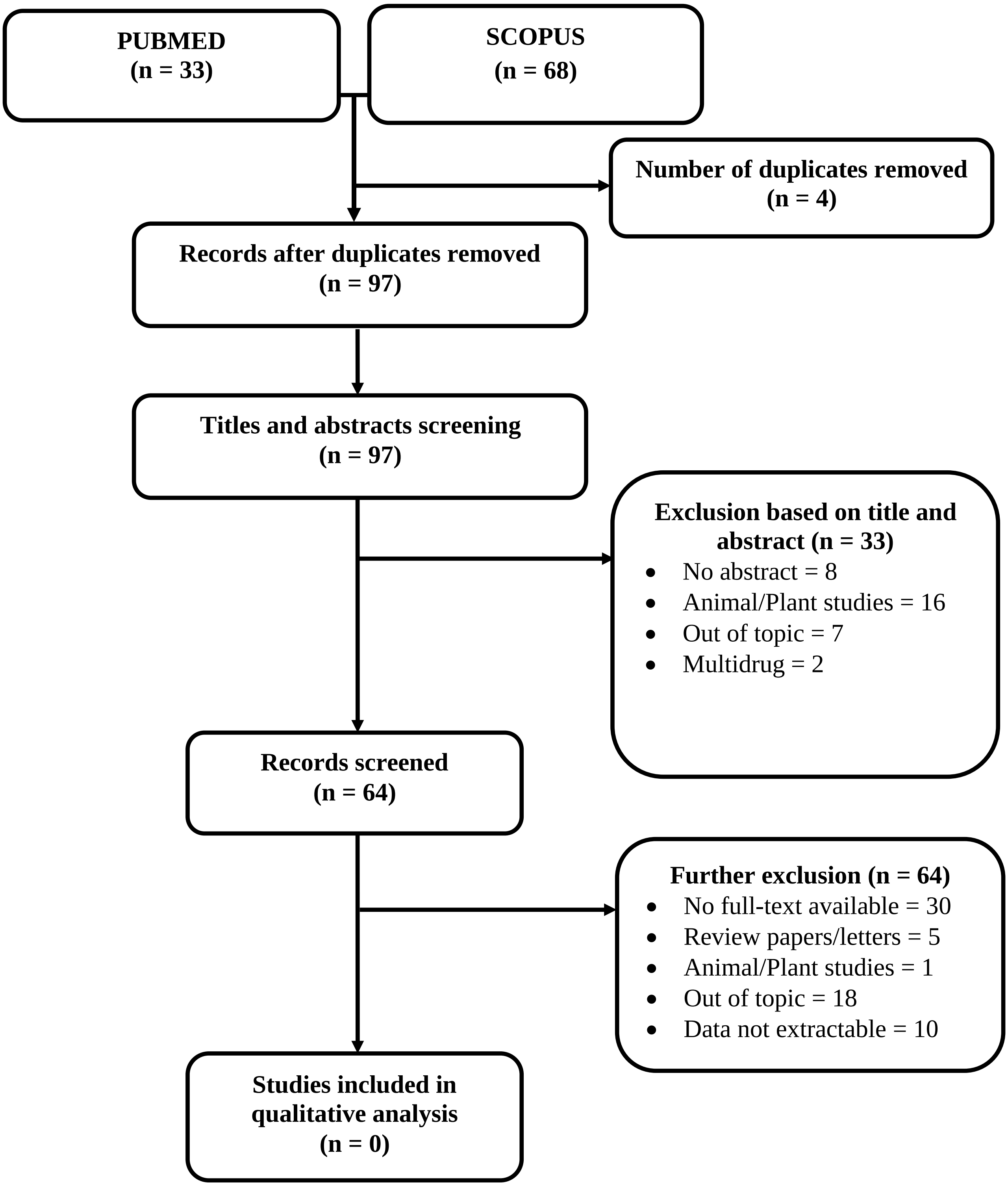

DISEASE: Leprosy
DRUG: Clofazimine

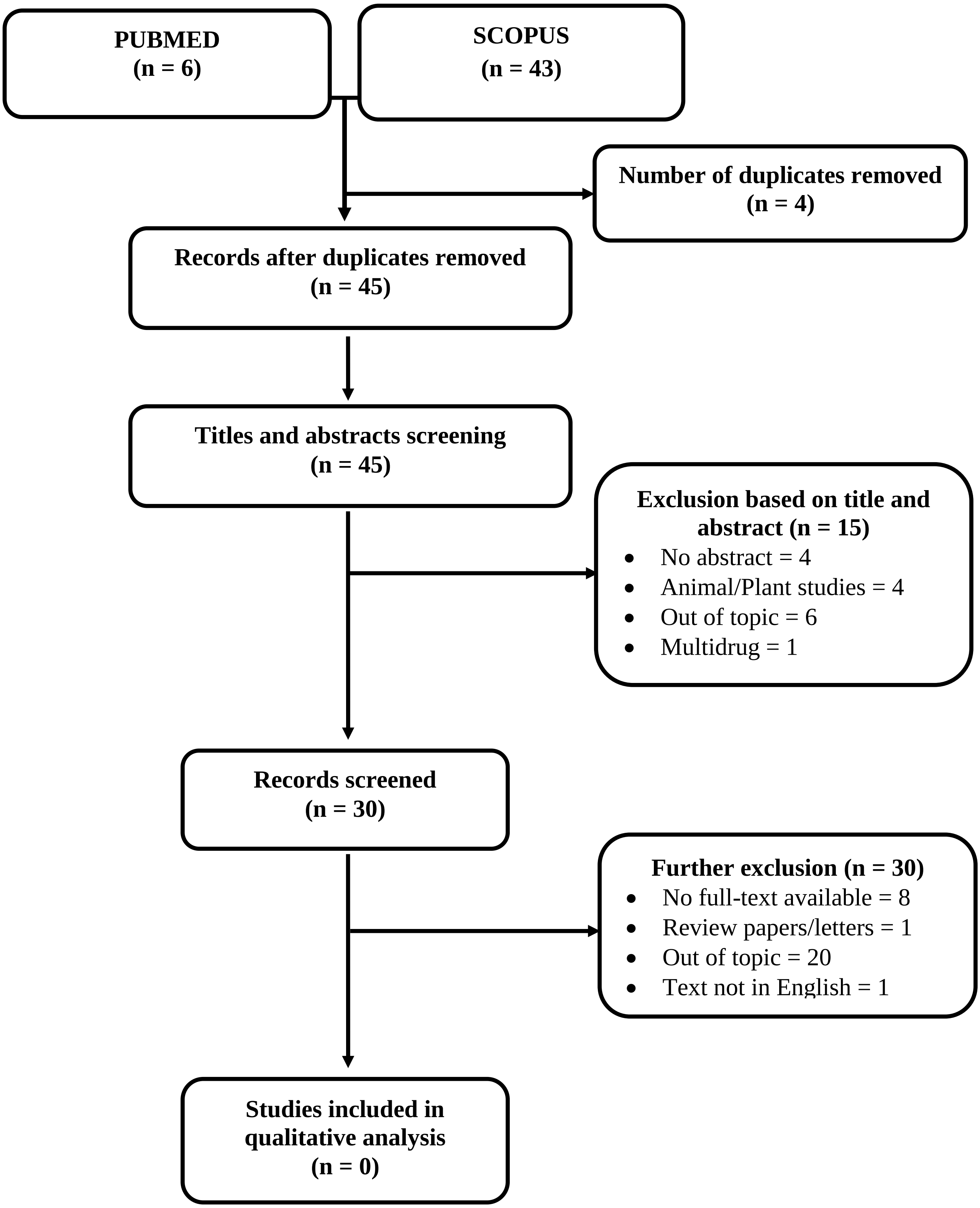

DISEASE: Leprosy
DRUG: Dapsone

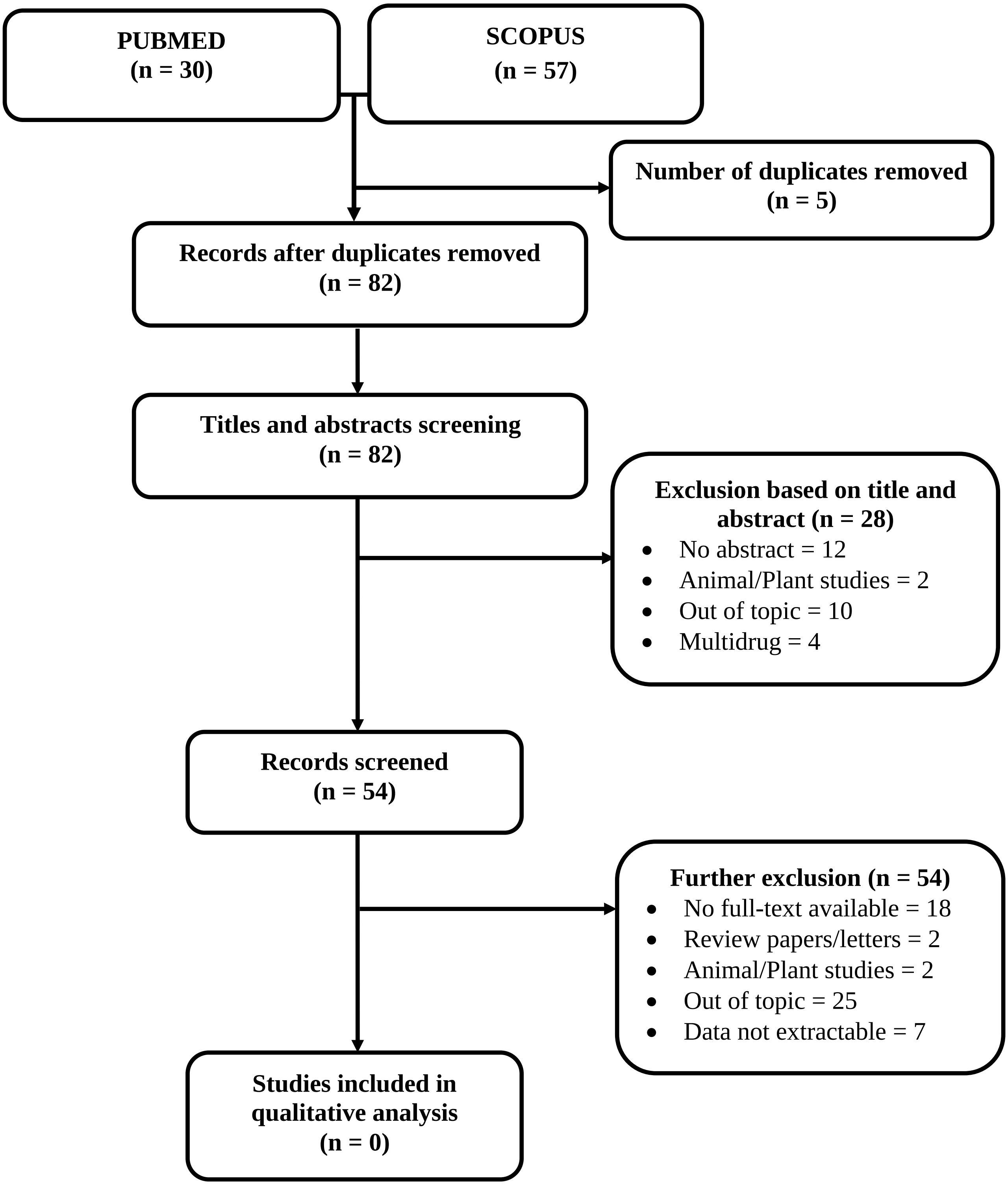

DISEASE: Trachoma
DRUG: Azithromycin

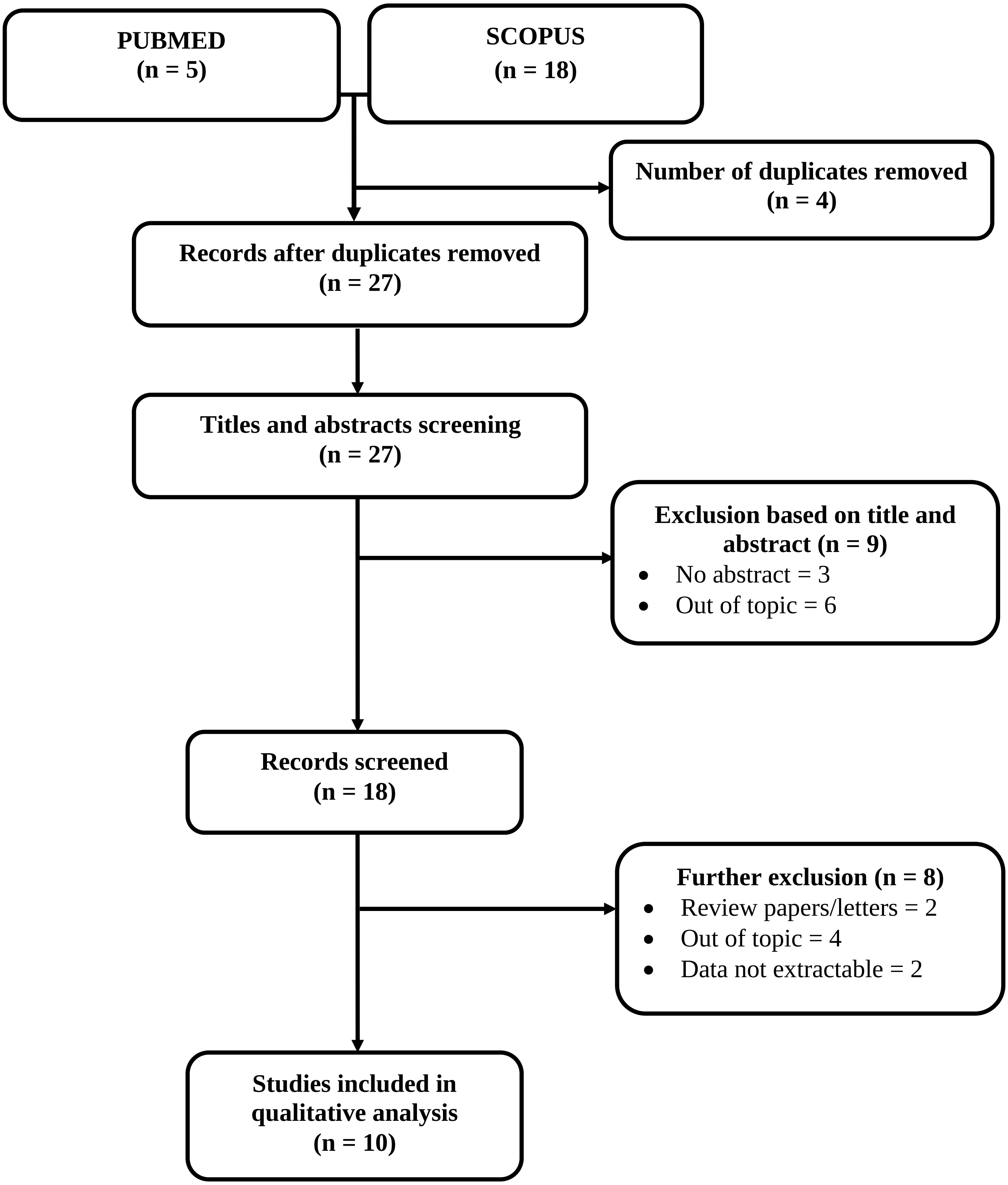

DISEASE: Taeniasis
DRUG: Praziquantel

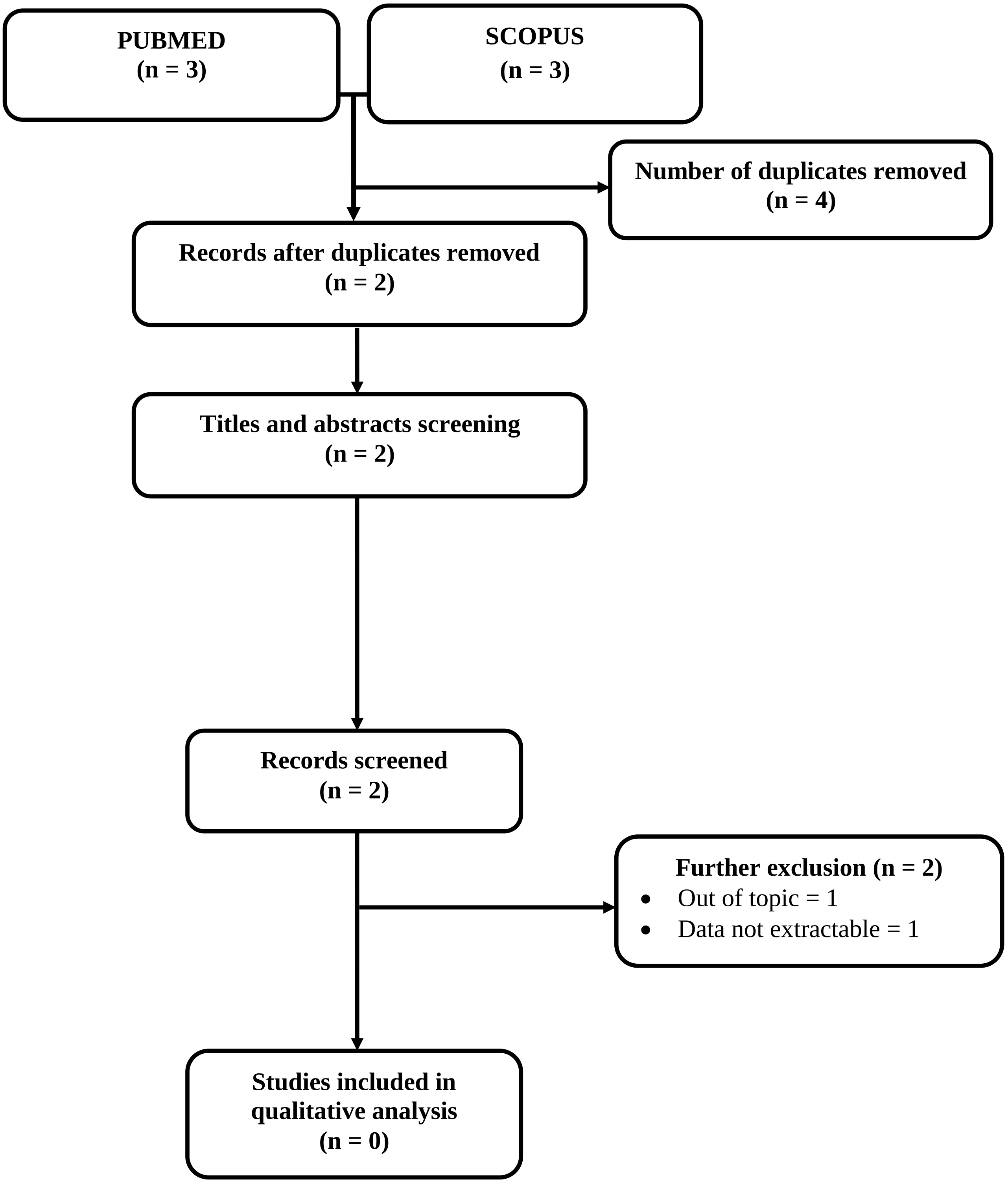

DISEASE: Taeniasis
DRUG: Niclosamide

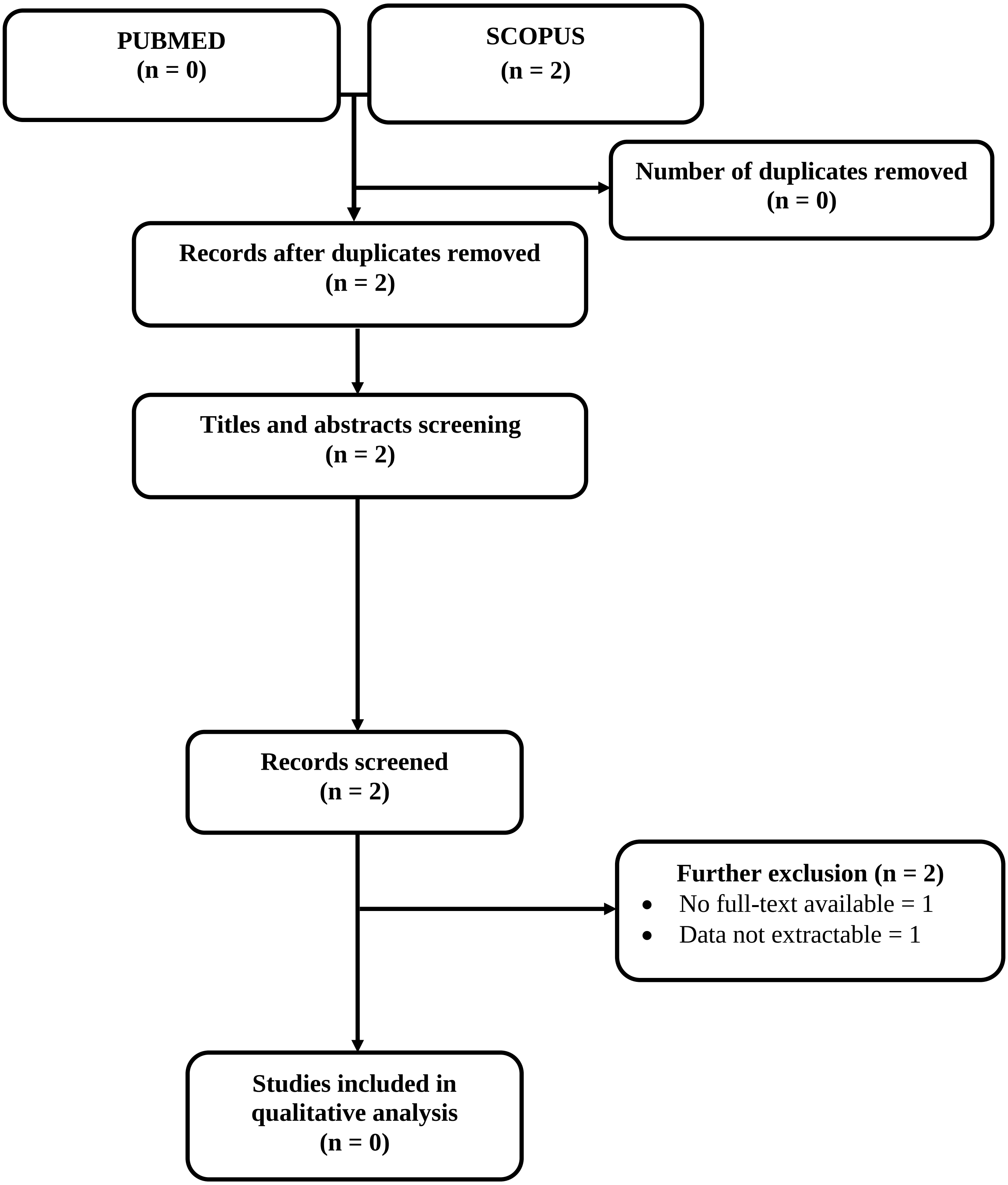

DISEASE: Trematodiasis
DRUG: Triclabendazole

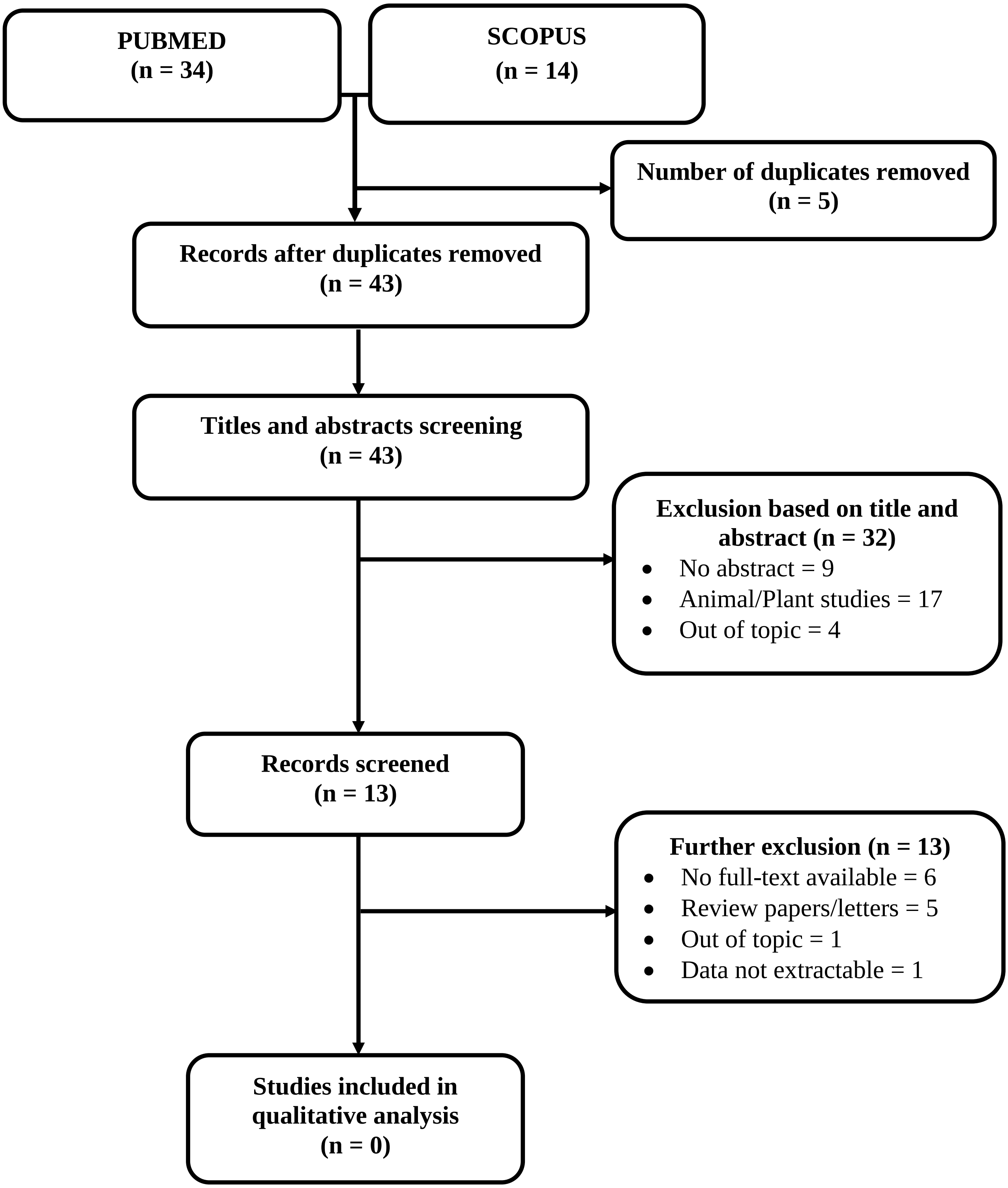

DISEASE: Lymphatic filariasis
DRUG: Albendazole

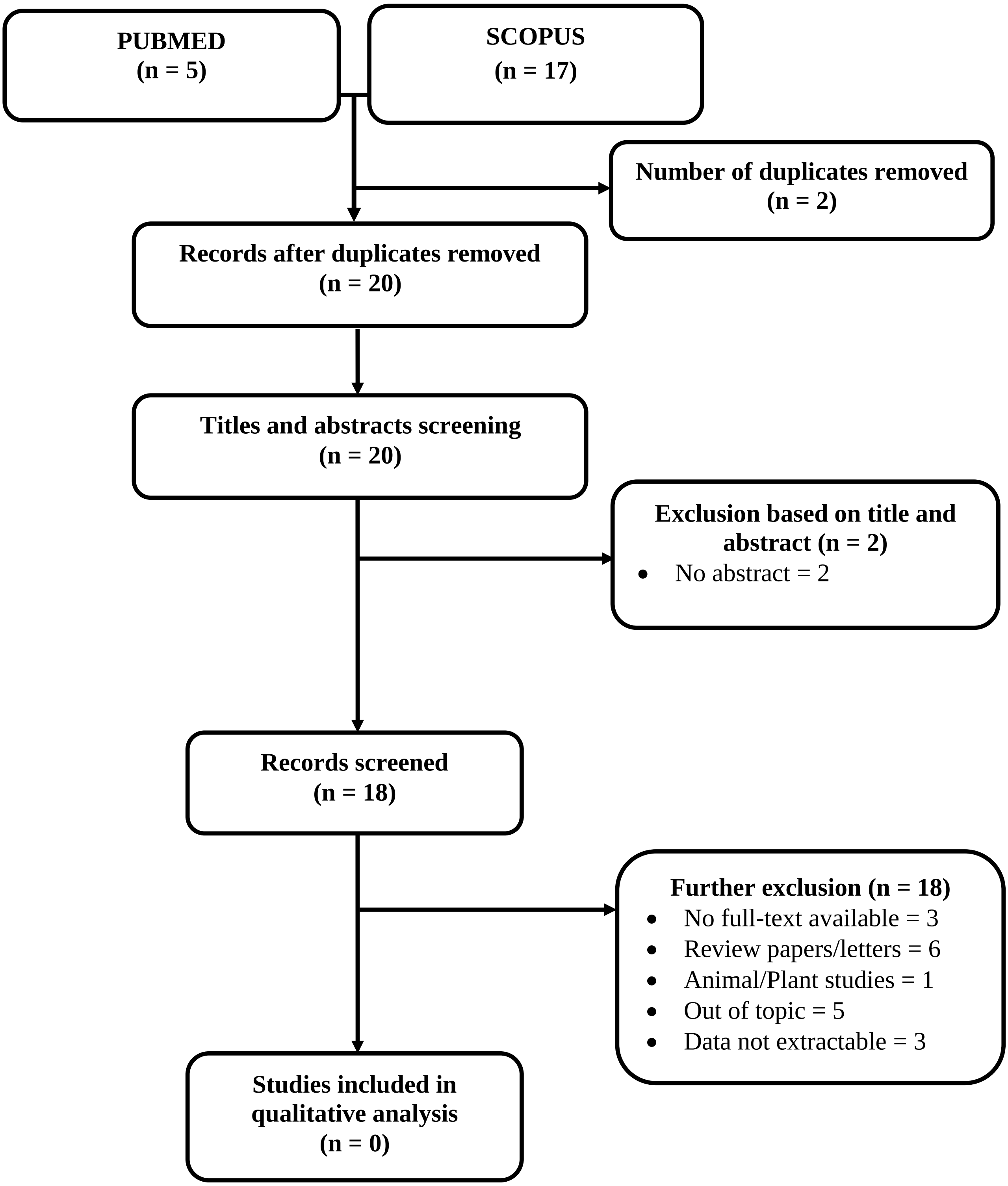

DISEASE: Lymphatic filariasis
DRUG: Diethycarbamazine (DEC)

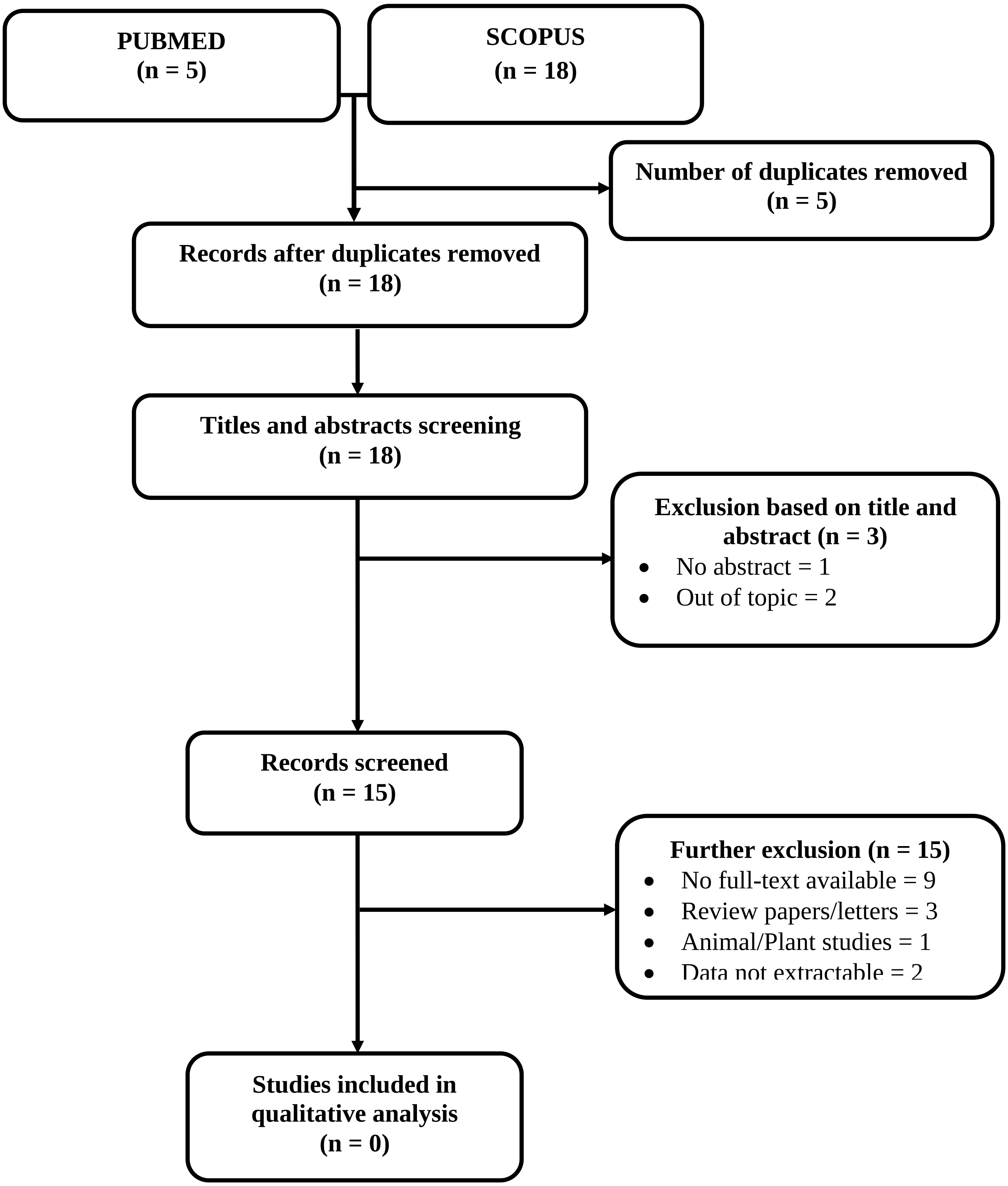

DISEASE: Lymphatic filariasis
DRUG: Ivermectin

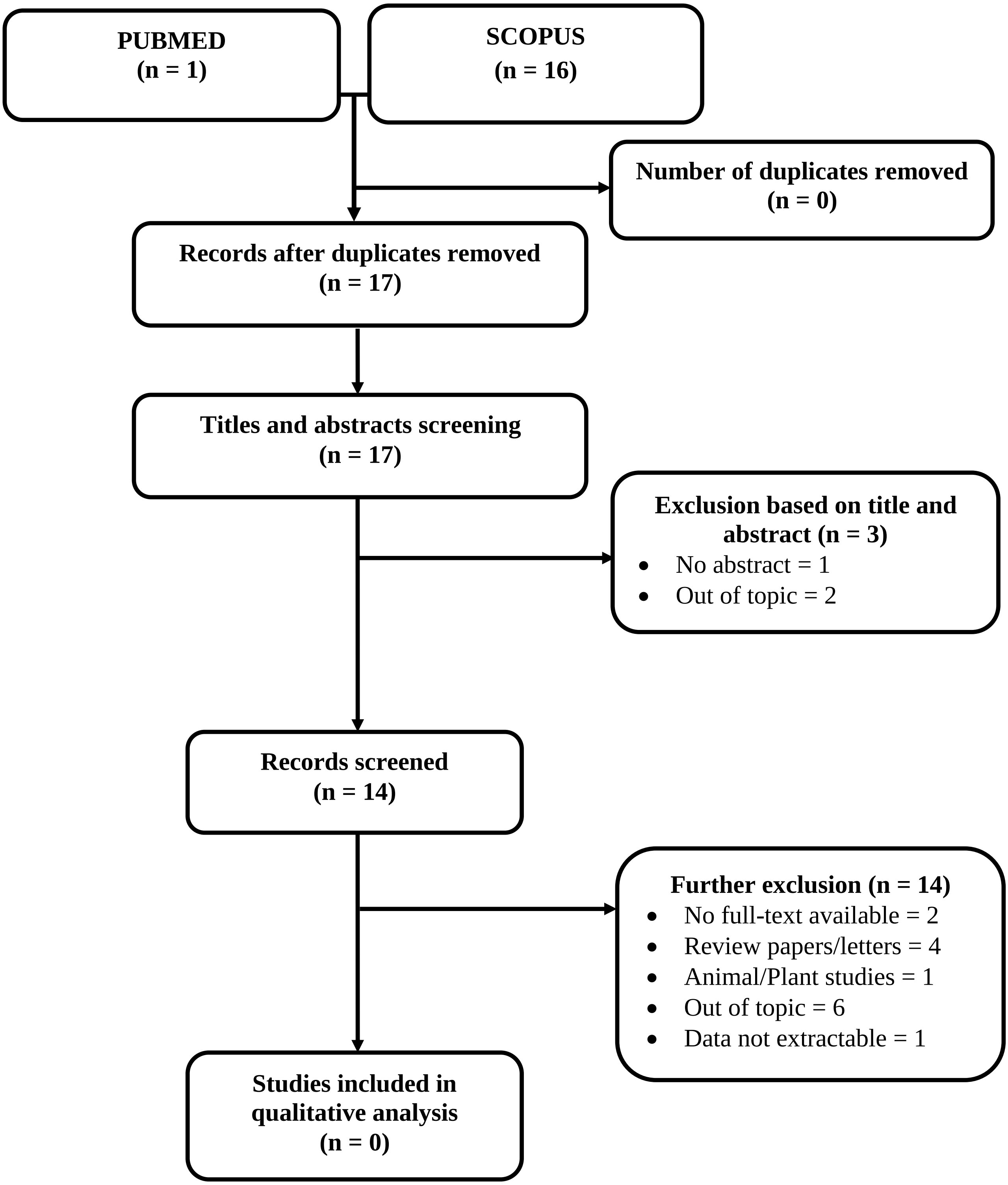

DISEASE: Onchocerciasis
DRUG: Ivermectin

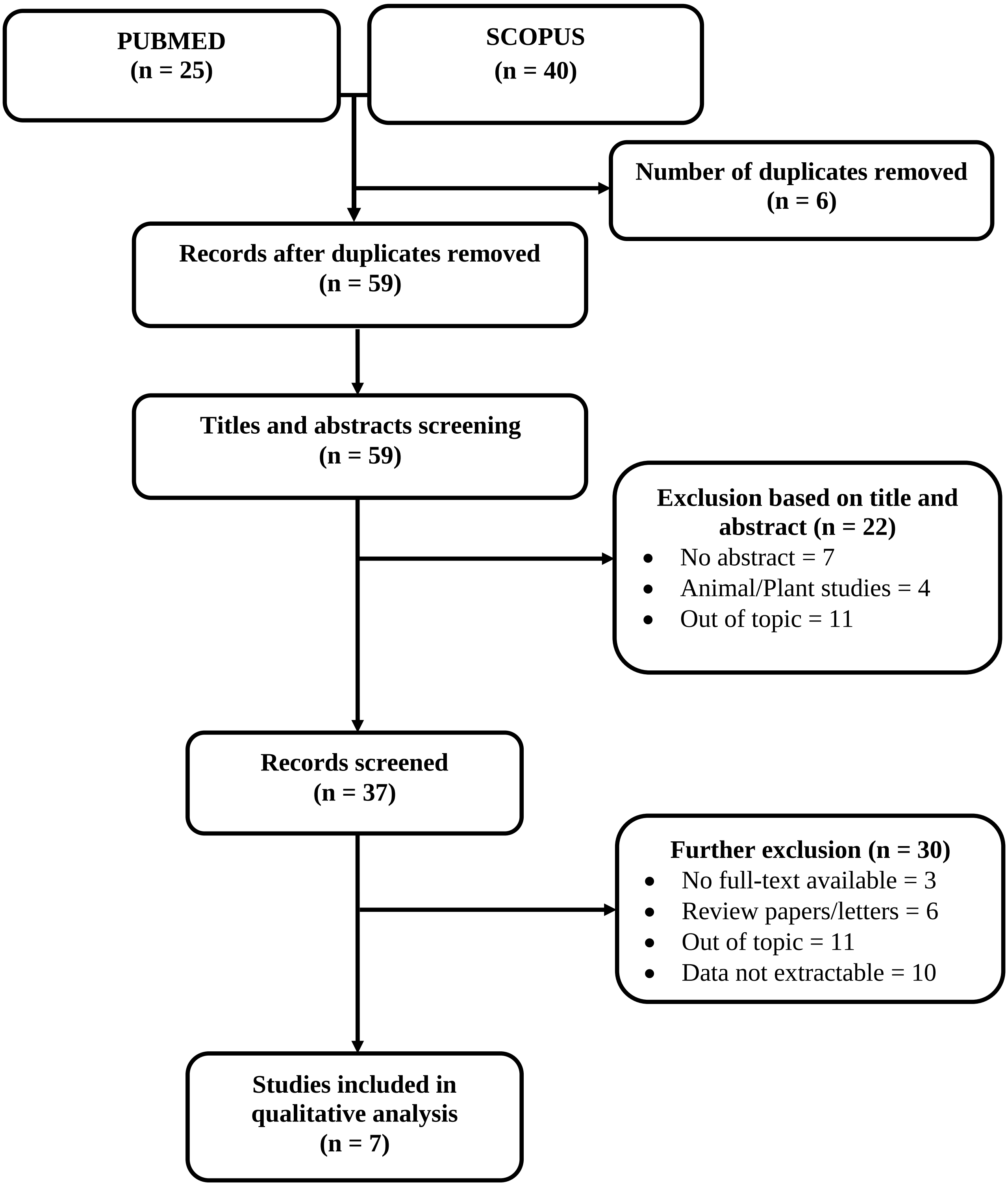

DISEASE: Schistosomiasis
DRUG: Praziquantel

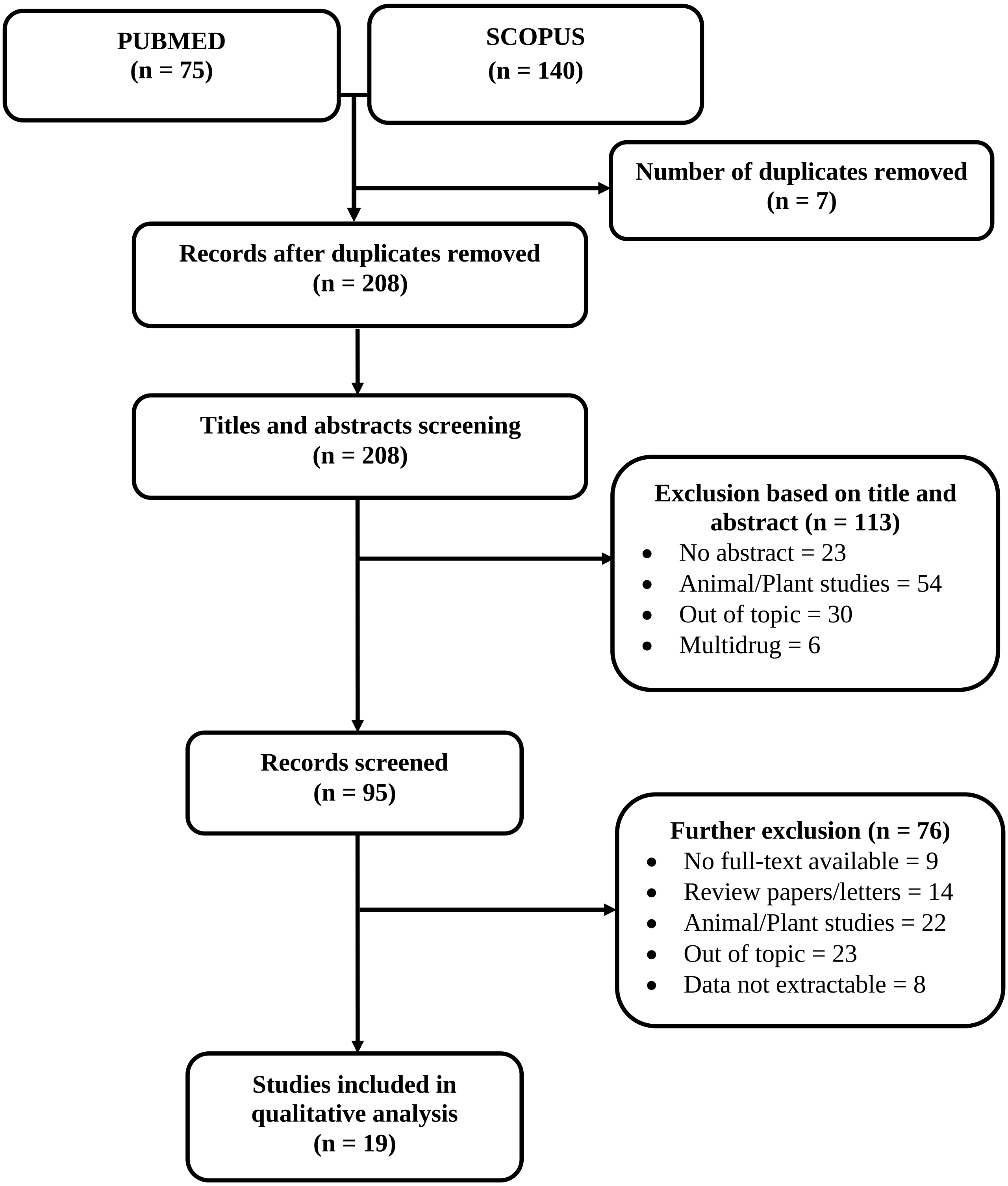

DISEASE: Soil-transmitted helminthes
DRUG: Mebendazole

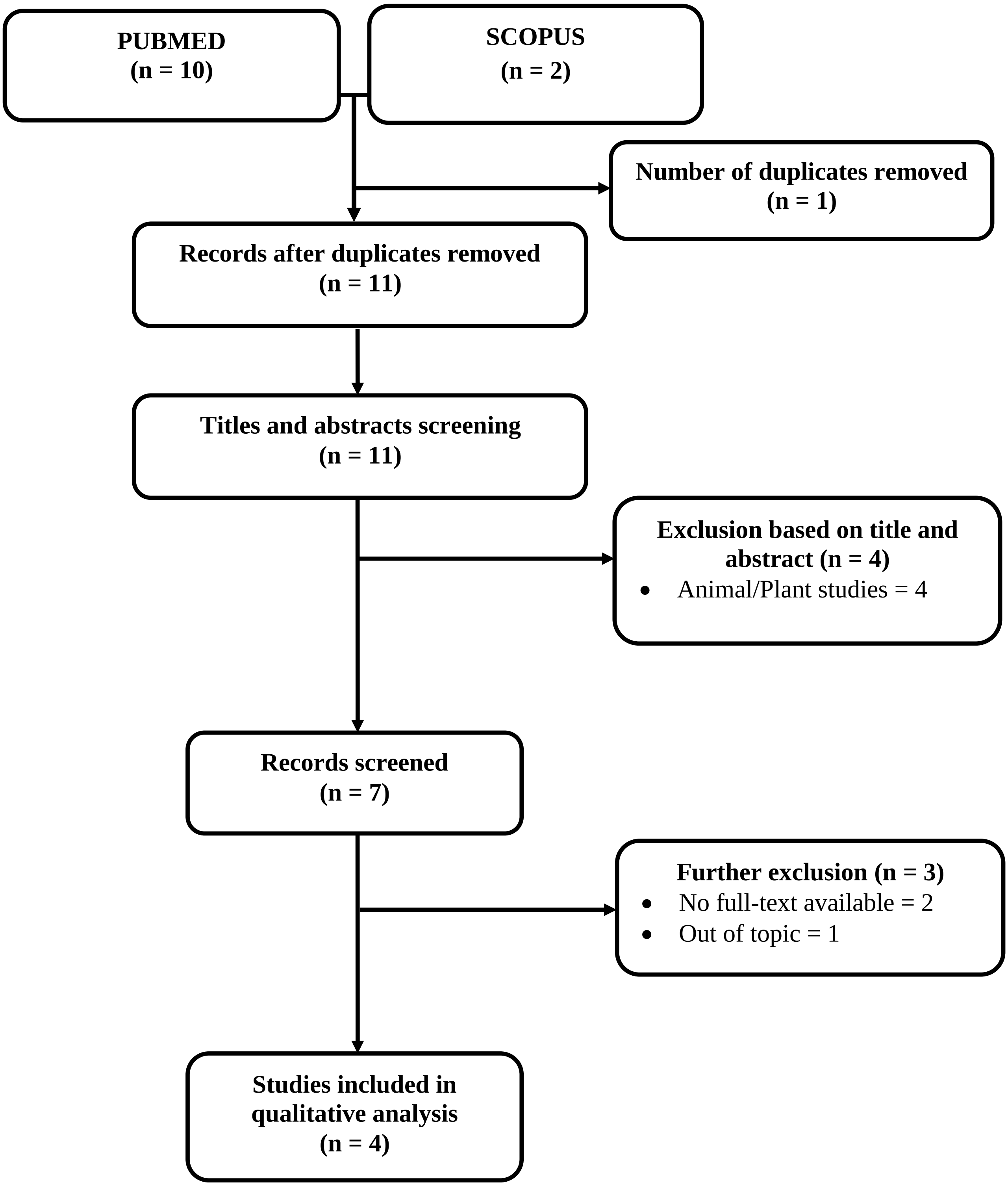

DISEASE: Soil-transmitted helminthes
DRUG: Albendazole

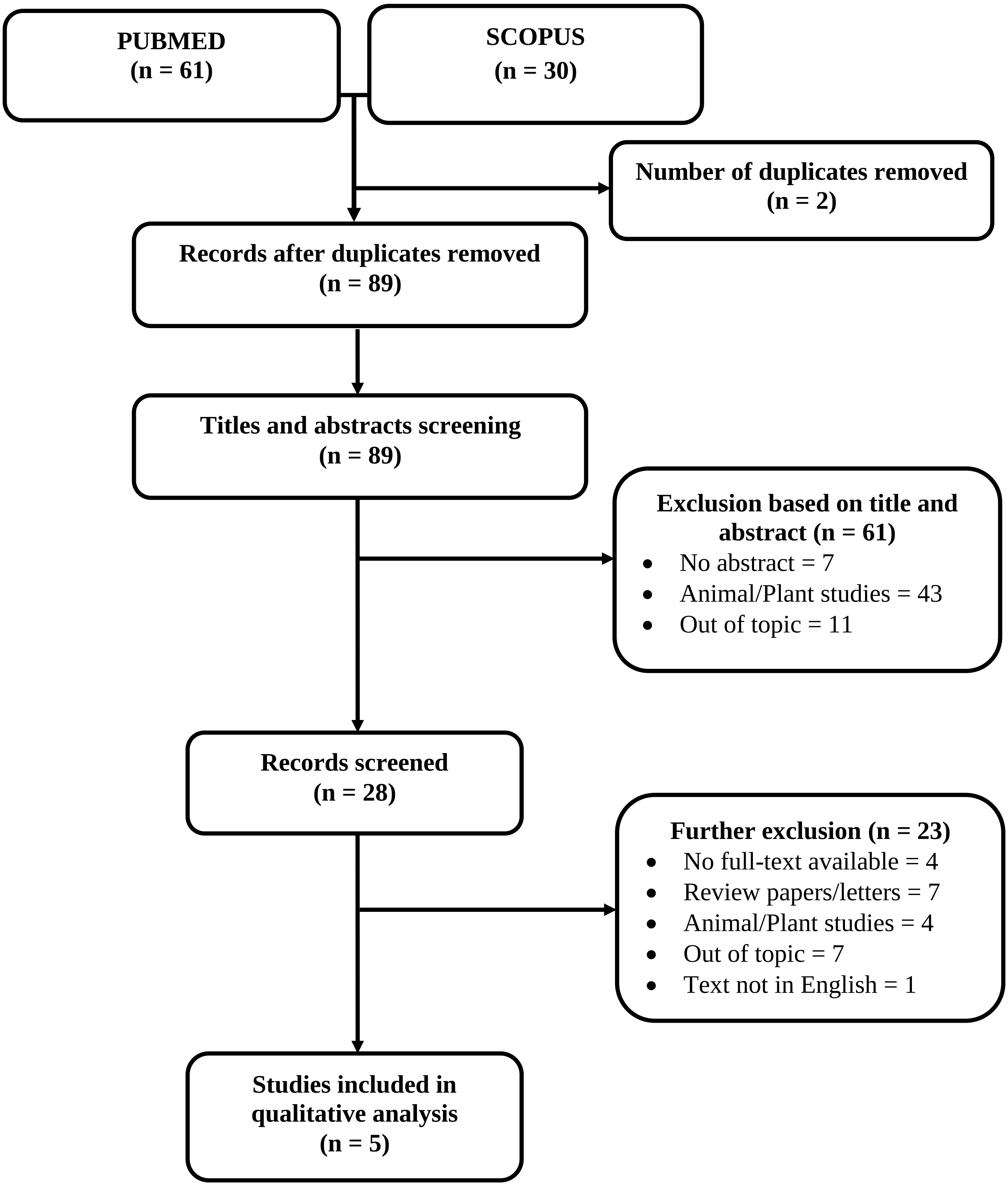

Supplement: Supplementary file 1 [file ijerph-16-01925-s001.zip › Supplementary files/Supplementary Materials S1.pdf]
