# Supplementary material for: Emerging Resistance of Neglected Tropical Diseases: A Scoping Review of the Literature
Source: Int J Environ Res Public Health. 2019 May 31;16(11):1925. doi: 10.3390/ijerph16111925 (PMC6603949; doi:10.3390/ijerph16111925)
Supplement: Supplementary file 1 [file ijerph-16-01925-s001.zip › Supplementary files/Table S2.pdf]

Table S2
NTDs search terms and search strategy

DISEASE	DRUGS	SEARCH TERMS	WEBSITE	NUMBER OF DOCUMENTS FOUND	NUMBER DOWNLOADED	SAVED TO ENDNOTE FOLDER
Chagas Disease	Nifurtimox	Chagas disease AND Drug Resistance AND nifurtimox	Pubmed and Scopus	69	69	Nifurtimox
Human African Trypanosomiasis	Suramin	Human African Trypanosomiasis AND Drug Resistance AND Suramin	Pubmed and Scopus	178	178	Suramin
	Eflornithine	Human African Trypanosomiasis AND Drug Resistance AND Eflornithine	Pubmed and Scopus	203	203	Eflornithine
	Melarsopol	Human African Trypanosomiasis AND Drug Resistance AND Melarsopol	Pubmed and Scopus	262	262	Melarsopol
	Pentamidine	Human African Trypanosomiasis AND Drug Resistance AND Pentamidine	Pubmed and Scopus	181	181	Pentamidine
Leishmaniasis	Amphotericin B	“Leishmaniasis” (MeSH) AND “Drug Resistance (MeSH)” AND “Amphotericin B (MeSH)”	Pubmed and Scopus	251	251	Amphotericin B
Leprosy	Rifampicin	“Leprosy (MeSH)” AND “Drug Resistance (MeSH)” AND “Rifampicin (MeSH)”	Pubmed and Scopus	332	332	Rifampicin
	Clofazimine	“Leprosy (MeSH)” AND “Drug Resistance (MeSH)” AND “Clofazimine (MeSH)”	Pubmed and Scopus	186	186	Clofazimine
	Daposne	“Leprosy (MeSH)” AND “Drug Resistance (MeSH)” AND “Daposne (MeSH)”	Pubmed and Scopus	516	516	Dapsone

DISEASE	DRUGS	SEARCH TERMS	WEBSITE	NUMBER OF DOCUMENTS FOUND	NUMBER DOWNLOADED	SAVED TO ENDNOTE FOLDER
Trachoma	Azithromycin	"Trachoma (Major)" AND "Drug Resistance" (MeSH) AND "Azithromycin (MeSH)"	Pubmed and Scopus	35	35	Azithromycin
Taeniasis	Praziquantel	"Taeniasis (Major)" AND "Drug Resistance (MeSH)" AND "Praziquantel (MeSH)"	Pubmed and Scopus	6	6	Praziquantel
	Niclosamide	"Taeniasis (Major)" AND "Drug Resistance (MeSH)" AND "Niclosamide (MeSH)"	Pubmed and Scopus	4	4	Niclosamide
Trematodiasis	Triclabendazole	"Trematodiasis (Major)" AND "Drug Resistance (MeSH)" AND "Triclabendazole (MeSH)"	Pubmed and Scopus	37	37	Triclabendazole
Lymphatic filariasis	Albendazole	"Elephantiasis, Filarial (Major)" AND "Drug Resistance (MeSH)" AND "Albendazole(MeSH)"	Pubmed and Scopus	20	20	Albendazole
	Ivermectin	"Elephantiasis, Filarial (Major)" AND "Drug Resistance (MeSH)" AND "Ivermection(Major)"	Pubmed and Scopus	17	17	Ivermectin
	Diethylcarbamazine (DEC)	"Elephantiasis, Filarial (Major)" AND "Drug Resistance (MeSH)" AND "Diethylcarbamazine (MeSH)"	Pubmed and Scopus	21	21	Diethylcarbamazine
Onchocerciasis	Ivermectin	"Onchocerciasis (MeSH)" AND "Drug Resistance (MeSH)" AND "Ivermectin (MeSH)"	Pubmed and Scopus	74	74	Onchoceriasis

DISEASE	DRUGS	SEARCH TERMS	WEBSITE	NUMBER OF DOCUMENTS FOUND	NUMBER DOWNLOADED	SAVED TO ENDNOTE FOLDER
Schistosomiasis	Praziquantel	“Schistosomiasis (MeSH)” AND “Drug Resistance (MeSH)” AND “Praziquantel (MeSH)”	Pubmed and Scopus	267	267	Praziquantel
Soil-transmitted helminthes	Mebendazole	“Helminthiasis (Major)” AND “Drug Resistance (MeSH)” AND “Mebendazole (MeSH)”	Pubmed and Scopus	30	30	Mebendazole
	Albendazole	“Helminthiasis (Major)” AND “Drug Resistance (MeSH)” AND “Albendazole (MeSH)”	Pubmed and Scopus	102	129	Albendazole
