# Supplementary material for: Emerging Resistance of Neglected Tropical Diseases: A Scoping Review of the Literature
Source: Int J Environ Res Public Health. 2019 May 31;16(11):1925. doi: 10.3390/ijerph16111925 (PMC6603949; doi:10.3390/ijerph16111925)
Supplement: Supplementary file 1 [file ijerph-16-01925-s001.zip › Supplementary files/Table S3.pdf]

Table S3a: Quality assessment of included studies

AUTHORS	DRUGS	YEAR OF PUBLICATION	STUDY DESIGN	BLINDING	SELECTION BIAS	WITHDRAWALS/ DROP-OUTS	CONFOUNDERS	DATA COLLECTION	DATA ANALYSIS	REPORTING	OVERALL
Kibona et al.	Suramin	2006	strong	weak	no rating	no rating	weak	moderate	weak	strong	weak
Darby et al.	Suramin	2008	weak	weak	no rating	no rating	weak	weak	weak	weak	weak
Ehrhardt et al.	Suramin	2006	weak	weak	no rating	no rating	weak	weak	weak	weak	weak
Leyon et al.	Suramin	2007	moderate	weak	weak	strong	weak	weak	strong	moderate	weak
Croft et al.	Suramin	2006	weak	weak	no rating	no rating	weak	weak	weak	weak	weak
Faust et al.	Suramin	2004	weak	weak	no rating	no rating	weak	weak	weak	weak	weak
Kuepfer et al.	Melarsopol	2012	moderate	weak	weak	strong	weak	weak	moderate	strong	weak
Likeufack et al.	Melarsopol	2006	strong	weak	weak	no rating	weak	weak	moderate	moderate	weak
Simarro et al.	Melarsopol	2006	moderate	weak	weak	strong	weak	moderate	weak	moderate	weak
Brun et al.	Melarsopol	2001	strong	weak	weak	weak	weak	weak	weak	moderate	weak
Pyana et al.	Melarsopol	2011	moderate	weak	moderate	strong	weak	moderate	weak	moderate	weak
Burri et al.	Melarsopol	2007	moderate	weak	weak	strong	weak	moderate	strong	moderate	weak
Bisser et al.	Melarsopol	2007	strong	weak	moderate	strong	strong	weak	strong	moderate	0
Matovu et al.	Melarsopol	2001	strong	moderate	weak	no rating	weak	moderate	weak	moderate	weak
Ruiz et al.	Melarsopol	2002	moderate	weak	strong	strong	weak	moderate	weak	moderate	weak
Eperon et al.	Melarsopol	2007	moderate	weak	strong	strong	strong	strong	strong	moderate	weak
Balasegaram et al.	Melarsopol	2006	moderate	weak	moderate	strong	moderate	moderate	strong	strong	moderate
Lejon et al.	Melarsopol	2008	strong	weak	moderate	moderate	weak	strong	strong	strong	weak
Blum et al.	Melarsopol	2001	moderate	weak	weak	strong	strong	strong	strong	moderate	weak
Kuepfer et al.	Melarsopol	2011	moderate	weak	moderate	strong	strong	strong	strong	strong	moderate
Schmid et al.	Melarsopol	2005	moderate	strong	strong	strong	strong	strong	strong	strong	strong
Kagira et al.	Melarsopol	2011	moderate	weak	weak	strong	strong	strong	strong	strong	moderate
Pepin et al.	Melarsopol	2005	moderate	weak	strong	strong	strong	strong	strong	strong	moderate
Pitto et al.	Eflornithine	2009	strong	moderate	moderate	moderate	strong	strong	strong	strong	strong
Truc et al.	Eflornithine	2012	moderate	weak	weak	strong	moderate	weak	weak	moderate	weak
Truc et al.	Eflornithine	2012	moderate	weak	weak	moderate	moderate	moderate	moderate	moderate	weak
Balasegaran et al.	Eflornithine	2006	moderate	weak	weak	strong	strong	strong	strong	strong	weak
Balasegaran et al.	Eflornithine	2009	moderate	weak	strong	weak	strong	strong	strong	strong	moderate
Chappuis et al.	Eflornithine	2005	moderate	weak	weak	strong	moderate	moderate	moderate	strong	moderate
Priotto et al.	Eflornithine	2008	moderate	weak	moderate	weak	moderate	strong	strong	strong	weak
Wengeer et al.	Eflornithine	2014	weak	weak	no rating	no rating	weak	weak	weak	weak	weak
Balasegaram et al.	Pentamidine	2006	moderate	weak	weak	strong	strong	moderate	strong	strong	weak
Pepin et al.	Pentamidine	2010	moderate	moderate	weak	strong	strong	strong	strong	strong	moderate
Lejon et al.	Pentamidine	2010	strong	weak	weak	strong	strong	strong	strong	strong	moderate
Jamonneau et al.	Pentamidine	2003	moderate	weak	weak	strong	strong	moderate	strong	strong	weak
Paul et al.	Pentamidine	2014	weak	weak	no rating	no rating	strong	weak	moderate	moderate	weak
Ruiz et al.	Pentamidine	2002	moderate	weak	moderate	strong	strong	moderate	strong	strong	weak
Lejon et al.	Pentamidine	2003	strong	weak	weak	moderate	strong	moderate	strong	strong	weak
Simarro et al.	Pentamidine	2006	strong	moderate	weak	moderate	strong	moderate	moderate	moderate	weak

Table S3b: Quality assessment of included studies

AUTHORS	DRUGS	YEAR OF PUBLICATION	STUDY DESIGN	BLINDING	SELECTION BIAS	WITHDRAWALS/ DROP-OUTS	CONFOUNDERS	DATA COLLECTION	DATA ANALYSIS	REPORTING	OVERALL
Truc et al.	Pentamidine	2012	moderate	weak	weak	strong	strong	moderate	strong	moderate	weak
Abel et al.	Pentamidine	2004	moderate	weak	weak	strong	moderate	moderate	moderate	moderate	weak
Buguet et al.	Pentamidine	2005	moderate	weak	weak	no rating	strong	moderate	weak	moderate	weak
Chappuis et al.	Pentamidine	2004	moderate	moderate	weak	moderate	moderate	moderate	strong	strong	weak
Eperon et al.	Pentamidine	2007	moderate	moderate	weak	strong	strong	moderate	strong	strong	weak
Clerinx et al.	Suramin	2012	moderate	weak	no rating	no rating	weak	weak	weak	weak	weak
Powar et al.	Suramin	2006	moderate	weak	no rating	no rating	weak	weak	weak	weak	weak
Pepin et al.	Eflornithine	2000	strong	moderate	weak	strong	strong	strong	strong	strong	moderate
Cherian et al.	Eflornithine	2010	moderate	weak	no rating	no rating	weak	weak	weak	weak	weak
Pepin et al.	Melarsopol	2006	strong	weak	weak	weak	strong	moderate	moderate	strong	weak
Sindato et al.	Melarsopol	2008	strong	weak	no rating	no rating	weak	weak	weak	weak	weak
Burri et al.	Melarsopol	2001	moderate	weak	no rating	no rating	weak	weak	weak	weak	weak
Diro et al.	Amphotericin B	2014	moderate	weak	weak	strong	strong	moderate	strong	strong	strong
Srivastava et al.	Amphotericin B	2011	moderate	weak	weak	no rating	weak	moderate	moderate	moderate	weak
Sundar et al.	Amphotericin B	2008	strong	strong	weak	strong	strong	moderate	strong	moderate	moderate
Sundar et al.	Amphotericin B	2003	strong	weak	weak	weak	strong	moderate	strong	strong	weak
Zhao et al.	Amphotericin B	2011	weak	weak	weak	no rating	weak	weak	weak	weak	weak
Sundar et al.	Amphotericin B	2014	strong	weak	weak	strong	strong	moderate	strong	strong	weak
Sundar et al.	Amphotericin B	2008	strong	weak	weak	no rating	strong	moderate	strong	strong	weak
Sinha et al.	Amphotericin B	2006	moderate	weak	weak	weak	weak	weak	weak	moderate	weak
Pimentel et al.	Amphotericin B	2011	weak	moderate	weak	no rating	weak	weak	weak	moderate	weak
Kumar et al.	Amphotericin B	2011	weak	weak	no rating	no rating	strong	weak	weak	moderate	weak
Omollo et al.	Amphotericin B	2011	strong	weak	weak	no rating	no rating	no rating	strong	moderate	weak
Couto et al.	Amphotericin B	2014	weak	weak	no rating	no rating	weak	weak	weak	moderate	weak
Bourguinat et al.	Ivermectin	2008	moderate	weak	moderate	no rating	strong	strong	strong	strong	moderate
Bourguinat et al.	Ivermectin	2007	strong	weak	weak	strong	strong	strong	strong	strong	moderate
Hoeraufet al.	Ivermectin	2008	strong	strong	weak	strong	strong	strong	strong	strong	moderate
Kudzi et al.	Ivermectin	2010	strong	weak	weak	strong	moderate	moderate	strong	moderate	weak
Ali et al.	Ivermectin	2002	moderate	weak	moderate	no rating	weak	moderate	moderate	strong	weak
Nana-Djeunga et al.	Ivermectin	2012	moderate	weak	weak	no rating	moderate	moderate	moderate	strong	weak
Osie-Atweneboana et al.	Ivermectin	2011	moderate	weak	moderate	moderate	strong	strong	strong	moderate	weak
Barakat et al.	Praziquantel	2011	moderate	weak	moderate	strong	moderate	strong	strong	moderate	weak
Botros et al.	Praziquantel	2005	strong	weak	moderate	weak	strong	strong	moderate	moderate	weak
Lawn et al.	Praziquantel	2003	moderate	weak	no rating	no rating	weak	weak	weak	weak	weak
Downs et al.	Praziquantel	2013	moderate	weak	weak	moderate	moderate	moderate	strong	strong	weak
Lelo et al.	Praziquantel	2014	moderate	weak	weak	no rating	moderate	strong	strong	strong	weak
Tweyongyere et al.	Praziquantel	2008	moderate	strong	moderate	weak	strong	strong	strong	strong	moderate
Tweyongyere et al.	Praziquantel	2009	moderate	strong	moderate	moderate	strong	strong	strong	strong	moderate

Table S3c: Quality assessment of included studies

AUTHORS	DRUGS	YEAR OF PUBLICATION	STUDY DESIGN	BLINDING	SELECTION BIAS	WITHDRAWALS/ DROP-OUTS	CONFOUNDERS	DATA COLLECTION	DATA ANALYSIS	REPORTING	OVERALL
Utzinger et al.	Praziquantel	2000	moderate	weak	moderate	no rating	strong	strong	moderate	strong	moderate
Yu et al.	Praziquantel	2001	moderate	weak	moderate	strong	strong	strong	strong	strong	moderate
N'Goran et al.	Praziquantel	2003	moderate	weak	weak	weak	moderate	strong	strong	strong	weak
Black et al.	Praziquantel	2009	moderate	weak	no rating	no rating	weak	weak	weak	weak	weak
Guidi et al.	Praziquantel	2010	moderate	weak	weak	moderate	moderate	moderate	strong	strong	weak
Lamberton et al.	Praziquantel	2010	moderate	weak	weak	no rating	moderate	strong	strong	strong	weak
Wang et al.	Praziquantel	2010	moderate	strong	moderate	weak	strong	strong	strong	strong	moderate
Ahmed et al.	Praziquantel	2012	moderate	strong	moderate	moderate	strong	strong	strong	strong	strong
Al-Sherbinyet al.	Praziquantel	2003	moderate	weak	moderate	no rating	strong	strong	moderate	strong	weak
Mwanakasale et al.	Praziquantel	2003	moderate	weak	moderate	strong	strong	strong	strong	strong	moderate
Raso et al.	Praziquantel	2004	moderate	weak	moderate	strong	strong	strong	strong	strong	moderate
Sheir et al.	Praziquantel	2001	moderate	weak	weak	weak	moderate	strong	strong	strong	weak
Vercruysse et al.	Albendazole	2011	strong	weak	strong	strong	strong	strong	strong	strong	moderate
Levecke et al.	Albendazole	2014	moderate	weak	moderate	moderate	weak	strong	strong	strong	weak
Diawara et al.	Albendazole	2009	strong	weak	weak	no rating	weak	weak	weak	moderate	weak
Diawara et al.	Albendazole	2013	moderate	weak	moderate	moderate	strong	strong	strong	strong	moderate
Albonico et al.	Mebendazole	2003	strong	strong	moderate	moderate	moderate	strong	strong	strong	strong
Albonico et al.	Mebendazole	2005	strong	strong	moderate	moderate	moderate	strong	strong	strong	strong
Lubis et al.	Albendazole	2012	moderate	weak	weak	weak	strong	moderate	moderate	strong	weak
Albonico et al.	Mebendazole	2002	moderate	weak	weak	weak	strong	moderate	moderate	strong	weak
Lubis et al.	Mebendazole	2012	moderate	weak	weak	weak	strong	moderate	moderate	strong	weak
Maher et al.	Azithromycin	2012	moderate	moderate	moderate	weak	weak	moderate	strong	strong	weak
Fry et al.	Azithromycin	2002	moderate	weak	moderate	strong	strong	strong	strong	strong	moderate
West et al.	Azithromycin	2014	strong	weak	weak	strong	weak	moderate	moderate	strong	weak
Keenan et al.	Azithromycin	2015	strong	weak	weak	weak	weak	moderate	strong	strong	weak
Haug et al.	Azithromycin	2010	strong	weak	weak	weak	strong	strong	strong	strong	weak
Coles et al.	Azithromycin	2013	moderate	weak	weak	weak	weak	moderate	strong	strong	weak
Batt et al.	Azithromycin	2003	strong	weak	weak	weak	weak	strong	strong	moderate	weak
Gaynor et al.	Azithromycin	2005	strong	weak	weak	weak	weak	moderate	strong	strong	weak
Gaynor et al.	Azithromycin	2003	strong	weak	weak	weak	strong	moderate	weak	strong	weak
Coles et al.	Azithromycin	2013	strong	weak	moderate	weak	strong	strong	strong	strong	weak
